# Importance of Genotype-Phenotype Correlation in the Population Screening of Familial Hypercholesterolemia

**DOI:** 10.7759/cureus.79252

**Published:** 2025-02-18

**Authors:** Tuhina Das, Saikat Mondal, Anup K Rawool, Swarnava Tarafdar, Anirban Ghosh

**Affiliations:** 1 General Medicine, All India Institute of Medical Sciences, Kalyani, Kalyani, IND; 2 Genetics, Sahaj Genetics Center, Pune, IND; 3 Radiodiagnosis, All India Institute of Medical Sciences, Kalyani, Kalyani, IND

**Keywords:** exon 9 mutation, homozygous familial hypercholesterolemia, low density lipoprotein receptor mutation, p.(ala431thr), tendon xanthoma

## Abstract

A 43-year-old male presented to our clinic with a complaint of multiple swellings on the extensor tendons of the elbows, ankles, and feet. On examination, he was found to have tendon xanthomas and xanthelasmas, and arcus lipoidosis. Investigations showed a low-density lipoprotein (LDL) level *of *214 mg/dl. He had been on statins for a decade. His pretreatment LDL was 226 mg/dl. His genetic workup showed a homozygous variant in exon 9 of the low-density lipoprotein receptor (LDLR) gene of chromosome 19 and was classified as having homozygous familial hypercholesterolemia. His treatment was intensified to the maximum tolerated dosage of statin and ezetimibe but LDL was far above the acceptable limit, so he was planned for monthly injections of proprotein convertase subtilisin/kexin type 9 protein (PCSK9) inhibitor, evolocumab.

Primary care physicians should have a keen eye on correlating the clinical and biochemical parameters of the patient with a genetic mutation analysis so as to not miss out on diseases with a rare occurrence such as homozygous familial hypercholesterolemia (HoFH) since early and optimal treatment with appropriate lipid-lowering therapies (LLT) is warranted to reduce the morbidity and mortality of patients. One should also increase awareness in the population for family planning due to increased maternal risk of atherosclerotic cardiovascular disease (ASCVD) during pregnancy, and also that children born to this population will have obligate heterozygous familial hypercholesterolemia (HeFH).

## Introduction

Familial hypercholesterolemia is a multifactorial autosomal dominant disease following Mendelian inheritance. Heterozygous familial hypercholesterolemia (HeFH) is more common with an incidence of 1 in 200-250 per million and the elevation of low-density lipoprotein (LDL) is less severe than in its counterpart, homozygous familial hypercholesterolemia (HoFH), which is rarer in occurrence [1]. It is classified based on the clinical phenotype and genetic correlation. Although there is no universal diagnostic guideline, the classifications commonly used are those of the Dutch Lipid Clinic, Simon Broome's, the American Heart Association, and the European Atherosclerosis Society criteria [1].

HoFH involves genetic mutations of the LDL receptor (LDLR)-mediated pathway, which results in high LDL cholesterol levels that respond poorly to medications that act by enhancing the LDL receptor activity, such as statins and ezetimibe. Most patients, if undiagnosed early, develop atherosclerotic cardiovascular disease (ASCVD) by the second decade of life. Hence, early diagnosis with mutation analysis, followed by aggressive management with conventional statins, ezetimibe, and proprotein convertase subtilisin/kexin type 9 protein (PCSK9) inhibitors like evolocumab is the mainstay of treatment.

Novel therapies independent of the LDLR activity - namely, microsomal triglyceride transfer protein (MTP) inhibitors such as lomitapide; antisense oligonucleotide inhibitors of apolipoprotein B (ApoB) such as mipomersen; and angiopoietin-3 (ANGPTL-3) antibody such as evinacumab - have shown to reduce LDL levels in such patients. LDL apheresis (LA), if initiated within the first decade of life, is crucial to the management of LDL levels.

At present, the decision to undergo genetic testing is solely based on the clinician's discretion, and therefore, it is of utmost importance to diagnose the clinical signs early and correlate them with any relevant familial history and LDL values, even if it is not conclusive [1].

## Case presentation

A 43-year-old male presented with a 27-year history of developing swellings in the right elbow which gradually developed at the ankles and over the metatarsophalangeal joints of the foot. He was initially diagnosed by traditional healers as having leprosy and was started on native remedies, which he continued for several years until his father’s sudden demise due to some cardiac ailments at the age of 72. His elder brother had a history of having some nonspecific chest pain and hypertension for which he was evaluated and started on lipid-lowering drugs and antihypertensives. The patient was evaluated by a local physician and found to have total cholesterol (TC) of 304 mg/dl, triglyceride (TG) of 185 mg/dl, high-density lipoprotein cholesterol (HDL-c) of 41 mg/dl, low-density lipoprotein cholesterol (LDL-c) of 226 mg/dl. He was started on atorvastatin 20 mg.

His family history did not have any telltale signs of familial hypercholesterolemia (FH). His mother and sister had no significant clinical features. The observed variations in lipid profile and the treatment undertaken by his family members are provided in Table 1.

**Table 1:** Serum cholesterol levels of the index case and his family members before and after treatment with LLTs. TC: total cholesterol; LDL: low-density lipoprotein; TG: triglyceride; HDL: high-density lipoprotein; LLT: lipd-lowering treatment

Relation	Age (in years)	Pretreatment Lipid profile (in mg/dl)				Treatment with dosage (in mg)	Posttreatment lipid levels (in mg/dl)			
		TC	LDL	TG	HDL		TC	LDL	TG	HDL
Proband	42	304	226	185	41	Rosuvastatin 40 + Ezetimibe 20 + Bempedoic acid 180	277	214	64	46
Mother	64	268	185	136	56	Rosuvastatin 20	198	129	124	44
Brother	47	316	207	274	54	Rosuvastatin 20 + Fenofibrate 160	276	176	206	59
Brother’s daughter	15	238	158	120	56	-	-	-	-	-
Sister	39	249	155	184	57	Rosuvastatin 20	230	150	153	49

**Figure 1:**
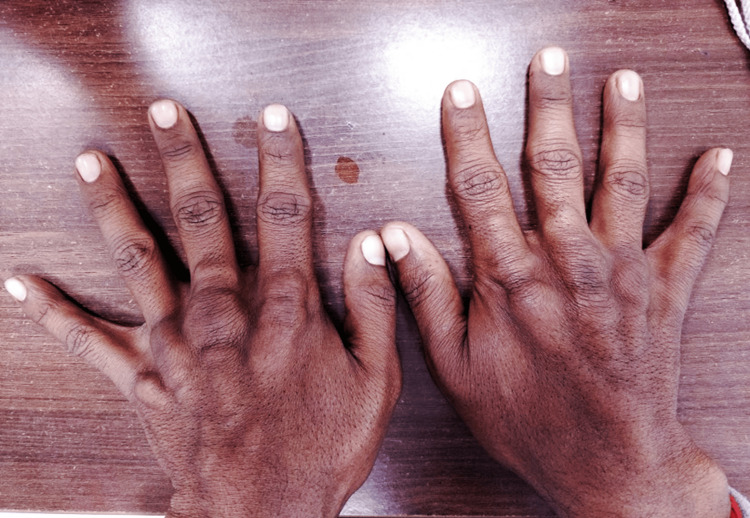
Tendon xanthomas of the hand

**Figure 2:**
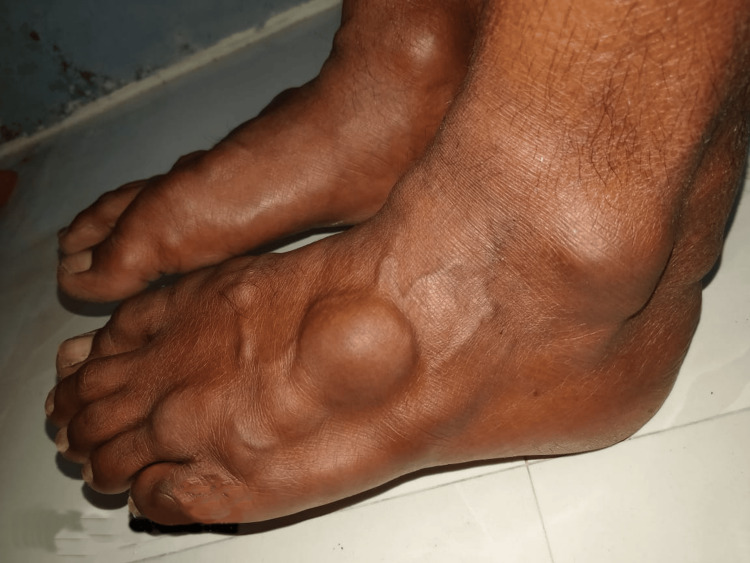
Tendon xanthomas of the feet

**Figure 3:**
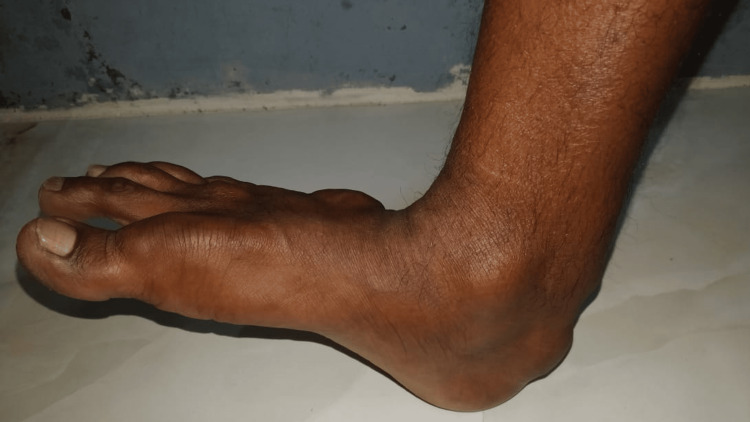
Tendon xanthomas at the Achilles tendon causing widening of the tendo-Achilles bilaterally

**Figure 4:**
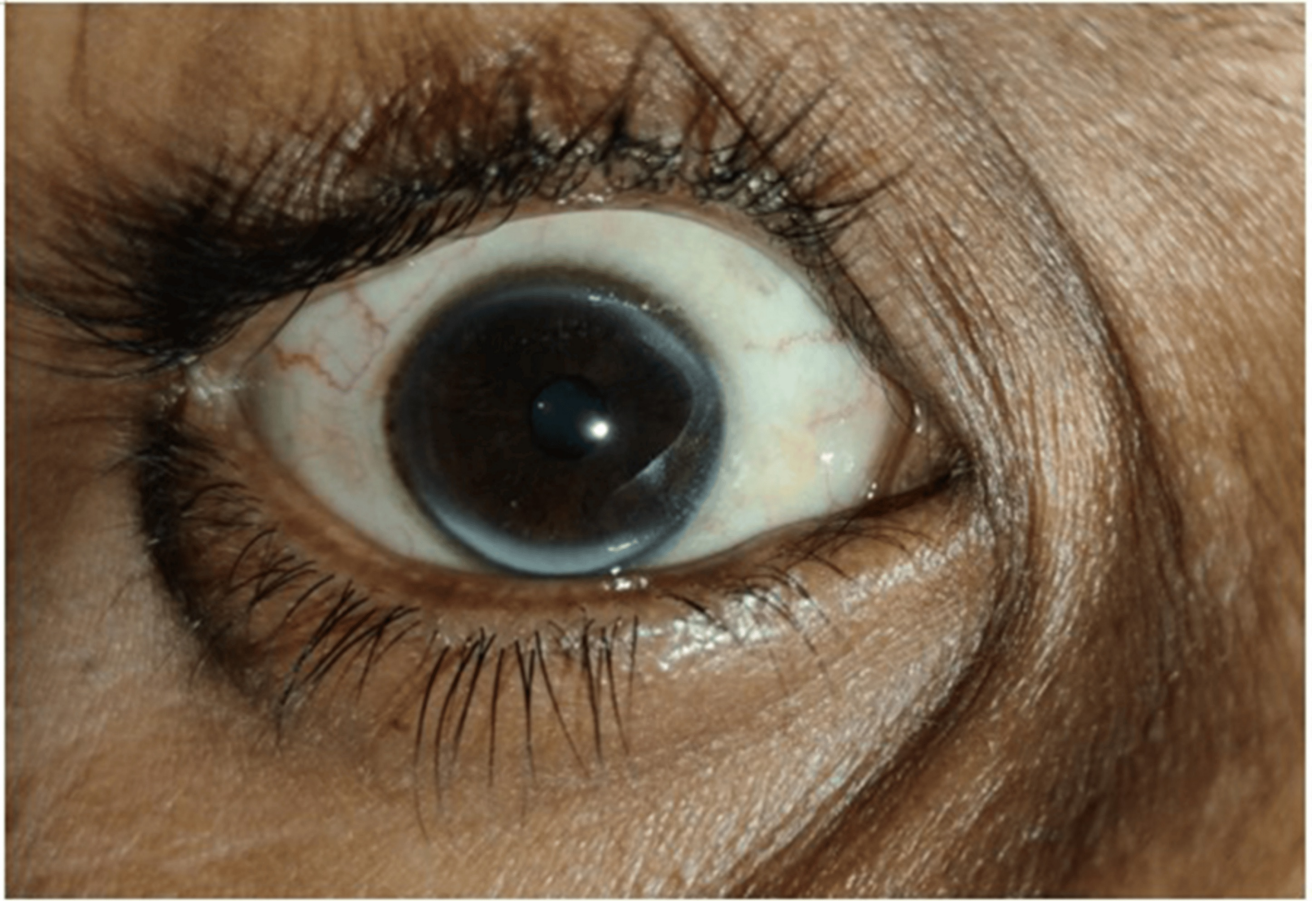
Incomplete arcus lipidosis

He presented to our clinic as he did not have any decrease in the size of the swellings even after continuing treatment for more than a decade. Examination revealed xanthelasmas around the elbow and firm to soft, non-tender, globoid swellings on the metacarpophalangeal (Figure 1) and metatarsophalangeal joints (Figure 2), and the elbows, and thickening and widening of the tendo-Achillies (Figure 3) as tendon xanthomas. He had partial arcus lipidosis (Figure 4) in both corneas.

**Figure 5:**
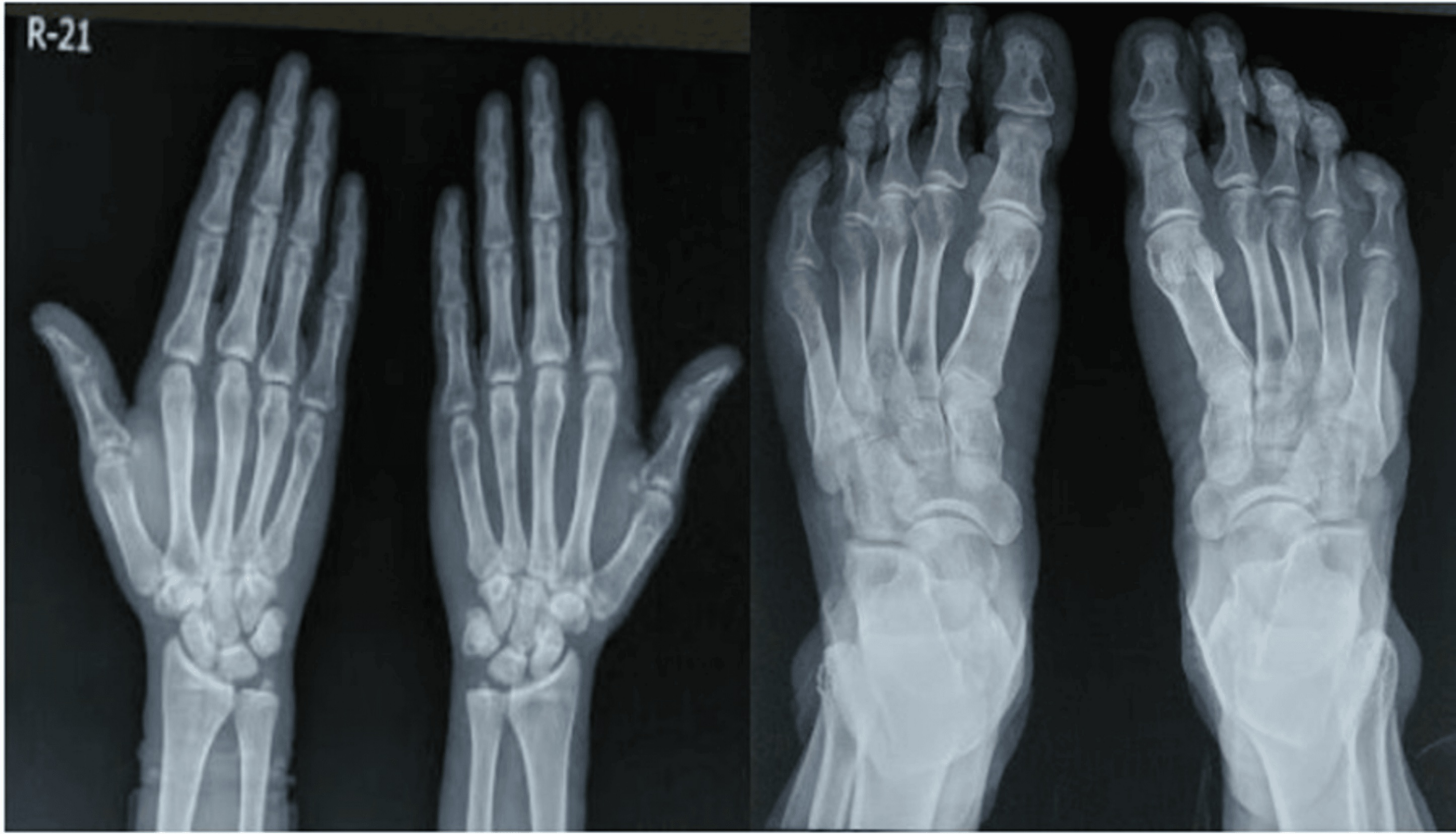
Radiographs of wrists (left) and feet (right) show no significant bony involvement at the sites of the tendon xanthomas.

Neurological examination was unremarkable. Biochemical investigations showed a TC of 478 mg/dl, TG of 420 mg/dl, HDL-c of 35 mg/dl, LDL-c of 214 mg/dl, very-low-density lipoprotein cholesterol (VLDL-c) of 22 mg/dl and an elevated lipoprotein-a (Lp-a) of 41.23 mg/dl. The thyroid profile was normal. A complete hemogram and peripheral blood smear showed no features of hemolytic anemia. Renal and liver functions were normal. Radiographic images of the wrists and ankles showed the absence of** **any significant bony involvement (Figure 5).

**Figure 6:**
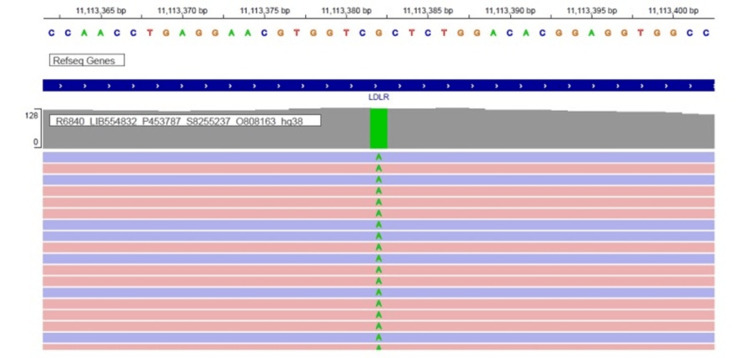
A novel homozygous mutation in exon 9 of the LDLR gene (chr19:g.11113382G>A;)

Excision biopsy was done for the swelling of the right elbow, a histopathological examination of which showed foam cells with cholesterol clefts supportive of xanthomas. His electrocardiogram showed preexcitation syndrome and the 2D echocardiogram showed concentric left ventricular hypertrophy, jerky septum, and an ejection fraction of 55%. The patient was planned for mutation analysis and targeted exome capture based re-sequencing of the relevant genes implicated previously in familial hypercholesterolemia and it demonstrated the exon 9 of the *LDLR *gene (chr19:g.11113382G>A;) having a homozygous mutation (Figure 6).

A single nucleotide base change from guanine to adenine caused the amino acid substitution from alanine to threonine at codon 431 (p.Ala431Thr; ENST00000558518.6). To understand the segregation patterns of alleles, recommendations were made to perform Sanger sequencing validation of the particular variant, identified from the next-generation sequencing (NGS) of parents and other family members of the proband so as to perform a cascade screening of their extended family. However, at the time of writing this report, the data was unavailable due to the financial constraints of the patient.

**Figure 7:**
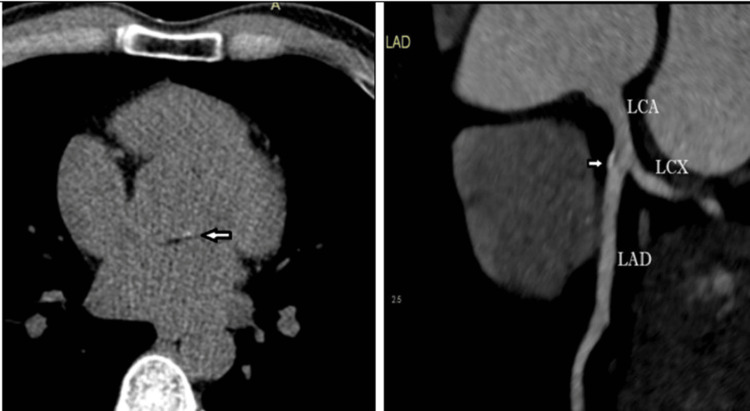
Plain images of CT showing (left) mild calcification at the aortic root (arrow). CT Coronary angiography image (right) showing mixed density plaque in ostioproximal LAD causing no significant stenosis. LCA: left coronary artery; LAD: left anterior descending artery; LCX: left circumflex artery

The patient underwent low-dose CT coronary angiography, which showed superficial bridging of the distal left anterior descending artery (LAD), mild calcification at the aortic root, and mixed-density plaque in the ostioproximal LAD causing no significant stenosis (Figure 7); no added intervention was deemed necessary at that time other than keeping the lipid levels under control.Carotid ultrasound Doppler imaging showed eccentric atheromatous plaque causing 50 percent lumen narrowing of the left carotid bulb.

The patient was started on rosuvastatin 40 mg, ezetimibe 20 mg, and bempedoic acid, and followed up every three months with his fasting lipid profile, but his LDL-c level did not reduce significantly. He was then planned for the initiation of PCSK9 inhibitor evolocumab, which is available in the country at present at select centers only, free of cost, to the patient.

## Discussion

HoFH has been reported worldwide at a frequency of 1 in 30,000 people [1] with a probability of <5% identification [2]. The prevalence of HoFH in India is approximately 1 in 490,000 [3]. The criteria for the diagnosis of HoFH according to the updated European Atherosclerosis Society of 2023 is either pretreatment LDL-c >500 mg/dl or LDL-c > 300 mg/dl after therapy, along with either cutaneous or tendon xanthomas before 10 years of age and/or untreated LDL-c indicative of heterozygous FH in both parents, although lower levels, especially in patients who received treatment or in children, do not exclude HoFH [4]. This should be confirmed by genetic affirmation of two mutant alleles involving LDLR,PCSK9, apolipoprotein B (ApoB) or LDLR adaptor protein 1 (LDLRAP1) gene locus or two or more such variants at different loci [4]. HoFH may also be associated with higher Lp(a) through an unknown mechanism not directly involved with the LDL receptor pathway [5]. Our patient had a pretreatment LDL-c value of less than 300 mg/dl, unlike the diagnostic criteria. Although his LDL-c levels did not fulfill the typical threshold for diagnosis, the possibility of HoFH remained due to the presence of other clinical features that needed confirmation by mutation analysis.

Barbosa et al. demonstrated that LDLR p.(Ala431Thr) causes disturbance of LDLR intramolecular interactions, affecting conformational stability of LDLR, leading to the impairment of the dissociation of LDLR-ApoB complex, hence reducing the capacity of LDL binding and its uptake [6]. This alteration leads to a defective β-propeller (YWTD-1) domain structure of the LDLR protein product [7]. β-propeller (YWTD-1) domain is involved in the folding of protein and recycling of the receptors [8]. However, they also showed with molecular dynamic simulations that the variant p.(Ala431Thr) did not significantly affect the stability of the protein structure and overall function of the protein product compared to other variants in important functional domains of the LDLR gene. Also, the patients with LDLR p.(Ala431Thr) variant had a milder phenotype compared to the familial hypercholesterolemia patients carrying LDLR null variants [6]. Hence, absolute values of LDL-c and clinical features should not be the sole diagnostic criteria for HoFH given the genetic variability and complexity of the disease.

Genetic mutation analysis of the observed variant has previously been reported as p.(A410T) in patients affected with hypercholesterolemia. In functional studies, the variant was reported to have considerably reduced LDLR activity compared to the wild-type protein and was found to be retained in the endosomal/lysosomal region, in contrast to the wild-type protein, which localizes on the cell surface [9]. The observed variant has been classified as Likely Pathogenic/Pathogenic by the ClinVar database [10]. The p.(Ala431Thr) mutation causes a conformational disturbance in LDLR, impairing LDL binding and uptake, though it does not have as severe an impact on protein stability as other mutations in critical domains [6].

The LDL receptor pathway primarily regulates the concentration of plasma cholesterol by receptor-mediated endocytosis into the cells. Monogenic FH is caused by several genetic defects encoding for proteins taking part in LDL uptake and its catabolism by the LDLR, ApoB, PCSK9, and LDLRAP1 [11]. LDLR is a transmembrane protein that is involved in the LDL-c uptake and its removal from circulation. More than 2,000 genetic variations have been documented in LDLR [12] and classified into five categories [13]. Class 5 variant of LDLR, as in the present mutant LDLR, is unable to recycle LDL into the endosome. The variant identified in this index case (p.Ala431Th) is previously described, and its occurrence in the epidermal growth factor precursor (EGFP) homology domain of the LDLR in previous studies demonstrated that up to 54% of LDLR variants resulting in FH were localized in the domain of EGF homology [14]. The ligand-binding domain positioning is done by this domain and it binds LDL on the cell surface. The acid-dependent dissociation of lipoproteins from the receptor in the endosome during receptor recycling is also performed by this domain [15]. p.(Ala431Th) may be related to a recycling-deficient phenotype as previously identified missense variants in this region were reported to be recycle-deficient.

A410T mutations have been reported previously in various ethnic groups [16]. Although it is a very common genetic disease, it is also one of the most underdiagnosed and inadequately treated disorders of lipid metabolism across the world [11]. Clinical suspicion should always be correlated with mutation analysis to confirm the diagnosis; earlier the treatment initiation, the lesser the disability burden on the patient and his family. Childhood screening (at less than 2 years old), if there is a history positive for premature ASCVD or hypercholesterolemia in the family, and possibly screening universally from 5 to 11 years as proposed by the current guidelines, might be beneficial for individual identification before the establishment of ASCVD [17]. Again, genetic differences in FH may likely impact individual sensitivity to lipid-lowering treatment (LLT). Dissimilarities in ASCVD related to genetic variations in FH might be partially explained due to the type of pathogenic mutations that change the responsiveness of a particular patient [18]. Also, screening of family members by “reverse cascade” testing should be encouraged. Early genetic screening in relatives through reverse cascade testing can significantly reduce the risk of cardiovascular complications and allow for timely interventions [19]. 

Having identified the underlying genetic mutation, we then explored its impact on the clinical phenotype and the treatment strategy necessary to address the patient's specific needs. Arcus lipidosis and cutaneous and tendinous xanthomas due to cholesterol deposits in skin and tendons are more common in HoFH, typically at an early age, although not exclusive to it. These might require surgical excision if cosmesis is required or there is a limitation in the range of mobility of the patient. Our patient developed tendon xanthomas in the second decade of life. Differentials for HoFH were cerebro-tendinous xanthomas and sitosterolemia, which were ruled out in our patient [4].

Early diagnosis and treatment are the essence of management to prevent ASCVD and early mortality. Low-dose CT coronary angiography is especially useful in detecting high-risk ostial plaques and valvular and supravalvular aortic disease which might progress even with reducing LDL-c levels and might need early intervention [4]. Although the maximum tolerated dose of statins, ezetimibe, and PCSK9 inhibitors such as evolocumab are the mainstay of treatment, they might not be quite effective due to their LDL receptor dependence. Most patients of HoFH need additional agents, namely, microsomal triglyceride transport protein inhibitors, such as Lomitapide, indicated only for HoFH; and injectable second-generation antisense oligonucleotides, such as mipomersen; or ANGPTL-3 antibodies such as evinacumab. However, our patient was not planned for the same as a first line of treatment due to lack of accessibility. Other modalities include LDL apheresis for reaching LDL-c targets <70 mg/dl in additional ASCVD risk factors such as elevated Lp(a) or diabetes mellitus or LDL-c <55 mg/dl in adults with clinical ASCVD [20]. Our patient did not reach the LDL targets with conventional treatment and was planned for monthly subcutaneous injections of evolocumab.

Prognostic indicators include age more than 30 years, males, history of ASCVD, elevated blood pressure, obesity, smoking, LDL-c >100 mg/dl, and Lp(a) > 50 mg/dl, and are independently linked to an increased hazard ratio of clinical ASCVD and, consequently, to increased morbidity and mortality [21].

## Conclusions

Though rare in occurrence, HoFH should be diagnosed early and aggressively treated to reduce the ASCVD burden. Clinical and biochemical parameters should be correlated with genetic assessment whenever feasible and awareness among healthcare workers and primary care physicians should be increased to identify the patients and their proper referral to specialist clinics at the earliest. While our patient did not meet the classical diagnostic threshold for HoFH based on LDL-c levels, the presence of a pathogenic LDLRmutation and characteristic clinical features supported the diagnosis. In light of the patient’s LDL receptor dysfunction, additional therapies such as lomitapide, mipomersen, or evinacumab are critical in optimizing cholesterol control and preventing cardiovascular complications. At present, the entire burden of evaluation and treatment is on the patient, and more and more national screening programs for the pediatric population and the availability of newer modalities of treatment to the patient at affordable cost are the need of the hour.

Genetic testing and reverse cascade screening in family members are critical for early diagnosis and intervention, potentially preventing future cardiovascular morbidity. HoFH is a lifelong condition requiring continuous management to reduce the risk of cardiovascular disease, and the patient warrants treatment, including novel therapies, as well as regular monitoring of cholesterol and cardiovascular status.
